# Renin-Angiotensin Activation and Oxidative Stress in Early Heart Failure with Preserved Ejection Fraction

**DOI:** 10.1155/2015/825027

**Published:** 2015-10-04

**Authors:** Smita I. Negi, Euy-Myoung Jeong, Irfan Shukrullah, Emir Veleder, Dean P. Jones, Tai-Hwang M. Fan, Sudhahar Varadarajan, Sergei M. Danilov, Tohru Fukai, Samuel C. Dudley

**Affiliations:** ^1^MedStar Washington Hospital Center, Georgetown University, Washington, DC 20010, USA; ^2^Cardiovascular Research Center and Cardiovascular Institute of Lifespan, The Warren Alpert Medical School, Brown University, Providence, RI 02903, USA; ^3^University of Illinois at Chicago, Chicago, IL 60612, USA; ^4^Division of Cardiology, Emory University School of Medicine, Atlanta, GA 30322, USA; ^5^Division of Pulmonary, Allergy and Critical Care Medicine, Emory University School of Medicine, Atlanta, GA 30322, USA; ^6^University of Tennessee, Memphis, TN 38163, USA

## Abstract

Animal models have suggested a role of renin-angiotensin system (RAS) activation and subsequent cardiac oxidation in heart failure with preserved ejection fraction (HFpEF). Nevertheless, RAS blockade has failed to show efficacy in treatment of HFpEF. We evaluated the role of RAS activation and subsequent systemic oxidation in HFpEF. Oxidative stress markers were compared in 50 subjects with and without early HFpEF. Derivatives of reactive oxidative metabolites (DROMs), F2-isoprostanes (IsoPs), and ratios of oxidized to reduced glutathione (*E*_*h*_ GSH) and cysteine (*E*_*h*_ CyS) were measured. Angiotensin converting enzyme (ACE) levels and activity were measured. On univariate analysis, HFpEF was associated with male sex (*p* = 0.04), higher body mass index (BMI) (*p* = 0.003), less oxidized *E*_*h*_ CyS (*p* = 0.001), lower DROMs (*p* = 0.02), and lower IsoP (*p* = 0.03). Higher BMI (OR: 1.3; 95% CI: 1.1–1.6) and less oxidized *E*_*h*_ CyS (OR: 1.2; 95% CI: 1.1–1.4) maintained associations with HFpEF on multivariate analysis. Though ACE levels were higher in early HFpEF (OR: 1.09; 95% CI: 1.01–1.05), ACE activity was similar to that in controls. HFpEF is not associated with significant systemic RAS activation or oxidative stress. This may explain the failure of RAS inhibitors to alter outcomes in HFpEF.

## 1. Introduction

Heart failure with preserved ejection fraction (HFpEF) accounts for up to 50% of heart failure (HF) cases [1, 2]. The prevalence of HFpEF is increasing [2], and nearly all patients with HF symptoms, including those with HF with reduced EF, have some component of HFpEF [3]. The pathogenesis of HFpEF is still incompletely understood. It is believed that before the advent of HF symptoms in HFpEF there is a latent phase of diastolic dysfunction (DD), associated with impaired left ventricular (LV) relaxation, elevated LV end diastolic pressure, and increased LV stiffness [4].

It has been shown that nitric oxide (NO) and nitric oxide synthase (NOS) have a role in cardiac relaxation, with a reduction in cardiac NO contributing to DD [5, 6]. The bioavailability of NO is dependent on the presence of reactive oxygen species (ROS) that oxidize NO and uncouple NOS, preventing NOS from producing NO [5]. Angiotensin II (Ang II) is known to cause uncoupling of NOS by activating NADPH (nicotinamide adenine dinucleotide phosphate) oxidase to produce ROS. This provides a possible link between RAS activation and DD [7]. Nevertheless, recent clinical trials have failed to show a benefit of RAS blockade in the treatment of DD [8–12].

Several convenient methods are available to measure oxidative stress in blood. Glutathione (GSH) is a major soluble intracellular peptide that eliminates peroxides and other oxidants [13]. GSH and its oxidized form (GSSG) can be reliably measured in plasma, and their ratio represents a redox couple, *E*_*h*_ GSH. Cysteine (CyS) comprises the major extracellular thiol, and, along with oxidized cysteine (CySS), it represents another measurable redox couple, *E*_*h*_ CyS [14]. Derivatives of reactive oxygen metabolites (DROMs) are a colorimetric assay for lipid peroxidation [15]. F2-isoprostanes (IsoPs) are a series of prostaglandin- (PG-) like compounds produced by the free radical-catalyzed peroxidation of arachidonic acid [16]. Recently, it has been shown that elevated levels of IsoPs are associated with incident cardiovascular events in patients with atrial fibrillation [17]. Additionally, we have used these assays in previous study to show increased systemic oxidative stress in patients with atrial fibrillation (AF) [18].

Preclinical studies have proposed that RAS and subsequent oxidation play a role in pathogenesis of DD in HFpEF. The cardiovascular effects of Ang II are believed to be because of its activation of NADPH oxidase [7]. Ang II also induces mitochondrial dysfunction, generating ROS such as superoxide (O_2_^∙−^). Overall, these are thought to lead to a reduction in NO bioavailability and a defect in myocardial relaxation [19]. Nevertheless, angiotensin convertase enzyme- (ACE-) inhibitors or angiotensin receptor blockers (ARBs) have not shown efficacy in treatment of DD [8–11]. This is in contrast to definite response seen with the use of RAS inhibitors in LV systolic HF [20, 21].

To help explain this paradox, we tested whether systemic RAS activation and associated oxidative stress were present in patients with DD in early HFpEF.

## 2. Methods

### 2.1. Study Design and Patient Recruitment

In a cross-sectional, case-control study, 50 subjects with NYHA functional Class I-II HF symptoms and echocardiographic evidence of HFpEF, as defined by preserved LV ejection fraction (EF) of >50% and abnormal echocardiographic LV relaxation pattern on pulsed-wave and tissue Doppler, and matched controls were recruited from the outpatient clinics and hospital at the Atlanta Veterans Affairs Medical Center and Emory University Hospital from July 2006 to February 2008 (https://www.clinicaltrials.gov/; NCT00142194). Cases and controls were matched for age in decades, smoking history, and diabetes mellitus, all known confounders in oxidative stress measurements. The protocol was approved by the Emory University Institutional Review Board. A written informed consent for participation in the study was obtained from all subjects.

Eligibility criteria for both cases and controls included age ≥18 years, an echocardiogram with mitral valve inflow velocities and tissue Doppler measurements within six months of enrollment, normal sinus rhythm, LV EF between 50 and 70%, and normal systolic and diastolic cardiac dimensions on qualifying echocardiogram. Exclusion criteria included systemic inflammatory disease, malignant neoplasm, severe valvular heart disease, HF NYHA Class III or IV, untreated hyper- or hypothyroidism, greater than mild cardiac hypertrophy, cardiomyopathy of any etiology, blood pressure (BP) > 180/100 mmHg on medications, concurrent illness resulting in life expectancy <1 year, and illicit drug or alcohol abuse.

### 2.2. Clinical Data

Demographic and clinical data were collected by review of medical records and physical examination upon enrollment. A qualifying standard 2D echocardiogram with Doppler examination was obtained at entry into the study. A single blood draw in a nonfasting state was obtained between 8:30 AM and 5:00 PM. Blood samples were collected from the antecubital vein and, for thiol measures, were immediately transferred to a microcentrifuge tube with 0.5 mL preservative solution of 100 mmol/L serine borate (pH 8.5), containing (per mL) 0.5 mg sodium heparin, 1 mg bathophenanthroline disulfonate sodium salt, and 2 mg iodoacetic acid. This was done to minimize autoxidation and hemolysis [22]. Samples were analyzed at the Emory Biomarkers Core Laboratory.

#### 2.2.1. Echocardiographic Data

All echocardiographic studies were performed with a GE System Vivid 7 Echocardiogram with the patient in left lateral position. Standard echocardiographic views were obtained per protocol. Left ventricular ejection fraction was calculated using the biplane modified Simpson rule. Cardiac inflow velocities were obtained by pulsed-wave (PW) Doppler analysis performed in the apical-4 chamber (A4C) plane. Peak early filling (*E*) and late atrial contraction (*A*) wave velocities were measured; the proportion of *E*/*A* waves was calculated. Mitral annular velocities were measured in the A4C plane at the septal and lateral mitral leaflet insertion sites using PW tissue Doppler imaging (TDI) [23]. The proportion of inflow/annular early velocities (*E*/*e*′) and annular early/annular late velocities (*e*′/*a*′) were calculated per standard guidelines. Isovolumic relaxation time and deceleration time were recorded. An independent cardiologist interpreted the studies using standard protocols [24].

### 2.3. Measurement of Oxidative Stress Markers

Markers used to measure systemic oxidative stress were the same as those we have characterized previously [18]: redox potential of the ratios of oxidized to reduced glutathione (*E*_*h*_ GSH) and cysteine (*E*_*h*_ CyS) in plasma (thiol ratios) [18], DROMs [15], and IsoPs [16]. The samples were stored at −80°C. Samples from cases and controls were treated identically. Laboratory technicians were blinded to the clinical data. The redox states (*E*_*h*_) of thiol/disulfide pools were calculated using Nernst equation:

(1) $$\begin{array}{c} E_{h}=E_{o}+\frac{RT}{nF}\ln\frac{\left[disulfide\right]}{{\left[thiol\right]}^{2}}, \end{array}$$

where *E*_*o*_ is the standard potential for redox couple, *R* is the gas constant, *T* is the absolute temperature, *n* is the number of electrons transferred, and *F* is Faraday constant. *E*_*o*_ used for glutathione and cysteine redox couples was −264 mV and −250 mV, respectively. Less negative *E*_*h*_ numbers implied a more oxidized state.

For measurement of IsoPs, plasma samples were acidified and a deuterated standard was added. This was followed by C-18 and Silica Sep-Pak extraction [16]. IsoPs were then converted to pentafluorobenzyl esters which were subjected to thin layer chromatography. F2-IsoPs were quantified by gas chromatography/mass spectrometry by using an Agilent 5973 MS with computer interference. After dissolution of serum in acidic buffer, an additive (N-N-diethyl-para-phenylenediamine) was added for DROM measurements [15]. Concentration of DROMs was determined through spectrometry (505 nm).

### 2.4. Measurement of RAS Activation

ACE activity and protein levels were analyzed in 31 (15 cases and 16 controls) subjects not taking any form of RAS inhibitor, since these are known to alter the measures [25–27]. Heparinized human plasma (20–40**** *μ*L) was diluted 1 : 5 parts with phosphate buffered saline (PBS) and incubated at 37°C with 200**** *μ*L of substrate for 2 hours. ACE activity was determined fluorometrically with two different substrates, Hip-His-Leu (HHL, 5** **mM) and Z-Phe-His-Leu (ZPHL, 2** **mM), and expressed as mU/mL [28, 29]. Levels of ACE protein were determined using plate precipitation assay based on a monoclonal antibody to the epitope localized on the N domain of ACE (9B9) and expressed as a percentage (%) of gold standard from pooled human plasma [25–27].

Western blot analysis was used to measure extracellular copper-zinc superoxide dismutase (ec-SOD) and the copper-delivering protein, ceruloplasmin (Cp), expression in representative samples from both groups. Briefly, plasma ec-SOD or Cp was concentrated by concanavalin-A sepharose chromatography, and protein expression was examined by immunoblotting with antibody against ec-SOD or ceruloplasmin (Dako Cytomation, Carpinteria, CA) [28, 29].

### 2.5. Statistical Analysis

Statistical analyses were performed using SAS software 9.1 (SAS Institute, Inc.). Sample size was based on a 0.90 power to detect the same difference that we observed in the least sensitive measure of oxidative stress in our previous study using a two-tailed *α*-level of 0.05 [18]. Baseline characteristics with normal distribution were compared between cases and controls using a paired *t*-test for continuous variables and Chi-square/Fisher exact test for categorical variables. Nonparametric tests were used for variables with skewed distribution. Mean (and median, where appropriate) levels of oxidative stress and ACE markers in cases and controls were compared using *t*-test for normally distributed variables and NPAR1WAY procedure (SAS software 9.1) for variables with skewed distribution. All variables significant on univariate analysis were entered into multiple logistic regression models to calculate adjusted odds ratios.

## 3. Results

We enrolled 50 patients with and without echocardiographic evidence of DD in HFpEF. The groups were well matched for known confounders in measurement of oxidative stress markers, including age (*p* = 0.96), smoking (*p* = 1.00), and diabetes mellitus (*p* = 0.77). The mean age of cases and controls was 64.8 ± 10.8 years (range: 45–83 years) and 65.0 ± 11.3 years (range: 43–88 years), respectively.

**Figure 1:**
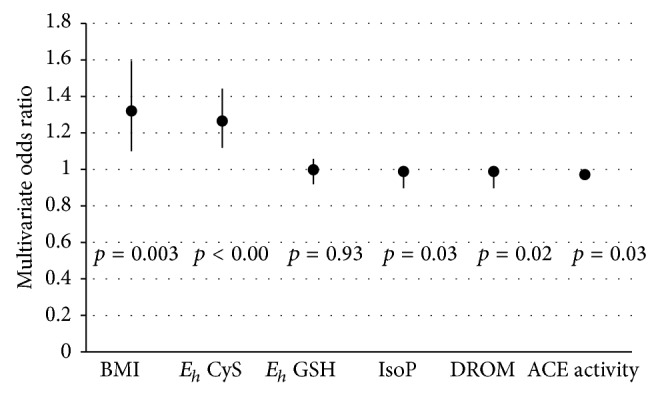
Multivariate odds ratios for association with diastolic dysfunction in early HFpEF. (BMI: body mass index; *E*_*h*_ CyS: redox potential of reduced to oxidized cysteine; *E*_*h*_ GSH: redox potential of reduced to oxidized glutathione; DROMs: derivatives of reactive oxygen metabolites; IsoPs: isoprostanes; ACE: angiotensin converting enzyme levels.)

Cases and controls only differed in male sex and BMI (Table 1). The association of higher BMI with DD was maintained on a multivariate analysis (*model 1*) using all demographic and clinical parameters as predictive variables (adjusted OR: 1.3; 95% CI: 1.1–1.6; Figure 1).

Table 2 compares markers for oxidative stress, ACE activity, and ACE protein levels in patients with and without DD. Three of four oxidative stress measures suggested that early DD was associated with a more reduced systemic oxidative state as compared to controls. *E*_*h*_ CyS was significantly more reduced (more negative) in patients with early DD (mean, −70.1 ± 7.8 mV; median, −72.9 mV in cases versus mean, −50.3 ± 11.6 mV; median, −50.6 mV in controls, *p* < 0.001). There was no significant difference in *E*_*h*_ GSH (mean, −118.8 ± 14.0 mV; median, −114.6 mV in cases versus mean, −118.4 ± 16.5 mV; median, −117.0 mV in controls, *p* = 0.93), a less sensitive measure of plasma redox state [23]. IsoPs levels (mean, 1495 ± 663 pg/mL; median, 1345 pg/mL in cases versus mean, 7385 ± 3241 pg/mL; median, 2341 pg/mL in controls, *p* = 0.03) and DROMs (mean, 375.2 ± 132.4 Carr units; median, 341.3 Carr units in cases versus mean, 474.5 ± 167.5 Carr units; median, 462.1 Carr units in controls, *p* = 0.02) were significantly lower in cases. The association between a more reduced *E*_*h*_ CyS and DD was maintained on a multivariate analysis (*model 2*) using gender, BMI, and oxidative stress measures as predictive variables (adjusted OR: 1.22; 95% CI: 1.08–1.37).

ACE activity, determined with HHL as substrate, demonstrated a mild but statistically insignificant increase in cases compared to controls (mean, 42.6 ± 9.6 mU/mL; median, 40.2 mU/mL in cases versus mean, 36.8 ± 9.0 mU/mL; median, 37.5 mU/mL in controls, *p* = 0.1). ACE protein levels were only marginally higher in patients with early DD (mean, 134.5 ± 38.2%; median, 112.8% in cases versus mean, 101.9 ± 22.2%; median, 104.3%; *p* = 0.03; adjusted OR: 1.05; 95% CI: 1.01–1.09).

**Figure 2:**
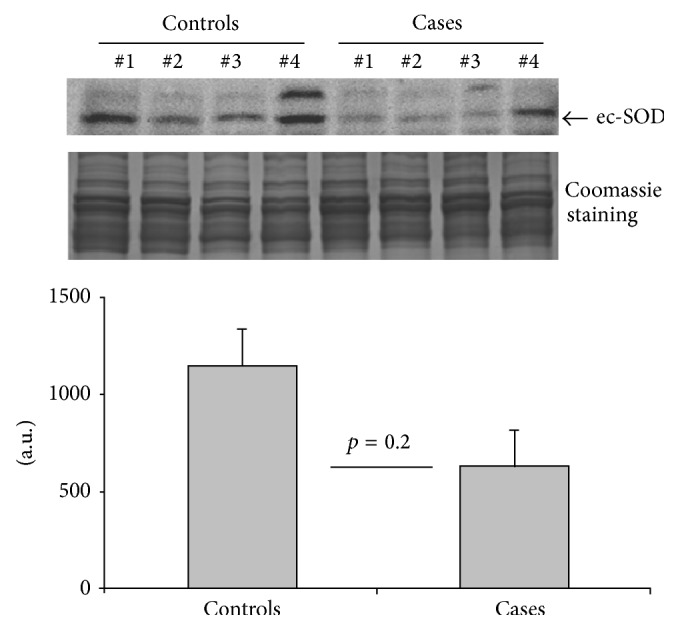
Protein expression of ec-SOD in blood samples from cases and control groups. The results depicted in the graph are presented as mean ± SE (cases (*n* = 6) and controls (*n* = 12)).

We measured ec-SOD in representative samples from both groups. Although there was a mild decrease in ec-SOD activity in cases, this did not reach statistical significance (*p* = 0.2) (Figure 2). Since ec-SOD is a copper enzyme, serum Cp, a marker protein for systemic copper, was also measured in the same samples but was not found to be altered in cases.

## 4. Discussion

In this study, we found no evidence of significant RAS activation or systemic oxidative stress in cases compared to matched controls. This is consistent with a lack of effect of RAS inhibitors and antioxidants in DD [8–12, 30]. Plasma ec-SOD activity and its copper-delivering protein, Cp, were not raised in cases. This also suggests a lack of increase in ROS, since systemic oxidative stress is known to upregulate peripheral ec-SOD activity [31]. Inhibition of ACE activity has been associated with elevated ec-SOD levels in studies [32], and unchanged ACE activity in the cases was consistent with unchanged ec-SOD.

There is a growing evidence that ROS signaling is compartmentalized [33]. It is possible that only local cardiac RAS activation or oxidation is required to generate DD. In a study by Inoue and group, peripheral oxidative stress was not detectable in HF after myocardial infarction (MI), even with progressive remodeling [34]. It was postulated that in the later phase of remodeling post-MI, ROS was generated mainly in the myocardium and multiple innate antioxidant defense mechanisms in the periphery stabilized levels of oxidative markers in blood and urine [34]. This might explain the contradiction between association of cardiac oxidative stress with DD and our findings. On the other hand, it is possible that systemic RAS activation and peripheral oxidative stress may occur late in the pathogenic cascade.

Alternatively, the lack of RAS activation and systemic oxidation may imply that other factors might be responsible for the onset of DD in HFpEF. Obesity has been associated with DD and may be one such factor [35, 36]. Our results are consistent with earlier studies showing correlation of obesity with DD [35, 36]. It has been shown that high BMI leads to a downregulation of adiponectin production in adipocytes [37]. Adiponectin, a member of complement factor C1q family, is believed to prevent endothelial injury in the heart and vasculature by multiple mechanisms, including promoting eNOS activity and preserving bioactive NO [38]. The lack of adiponectin in obesity may lead to progression of HFpEF [39]. Cardiac oxidation may be concentrated in obese individuals because of increased epicardial adipose tissue (EAT) [40, 41]. EAT is a source of adipocytokines that have both apocrine and paracrine effects on adjacent myocardial cells leading to chronic local and systemic inflammation [42].

Few recent studies have found an association of systemic oxidative stress with HFpEF. In their study [43–45], Vitiello et al. measured plasma levels of C-reactive protein, interleukin-6, 8-epi-prostaglandin F2*α*, and thiobarbituric acid reactive substances (TBARS) in eighteen HFpEF patients and 14 controls. The authors concluded that HFpEF exhibits an elevation in a broad spectrum of biomarkers indicative of an inflammatory and a prooxidative state [43]. Of note, however, in their study, the HFpEF population was older by a decade than the controls. Aging, by itself, can increase oxidative stress and its markers [46]. Our study, on the other hand, has shown that oxidative stress and angiotensin activity were not elevated in HFpEF. The results of our study support the lack of clinical benefit seen with therapies to reduce angiotensin activity and oxidative stress such as RAAS inhibitors in HFpEF in large clinical trials [8–12].

### 4.1. Limitations and Future Directions

There are limitations to our study. Foremost, despite consistency with previous clinical trials, it is possible that our study did not have sufficient power to detect associations between RAS or oxidative stress and HFpEF. On the other hand, a similar sized study easily detected a difference in oxidative stress markers in patients with and without AF [18]. Blood samples were drawn from nonfasting subjects and at variable times during the day. Thiol reduced state is affected by meals in animals [47]. Nevertheless, variations are relatively small and plasma levels of DROMs and IsoPs are not affected by the prandial state [48]. Also, it is known that plasma levels of oxidized and reduced thiols undergo a small diurnal variation [49]. Again, DROMs and IsoPs are not known to show any diurnal variation [18]. Moreover, four different markers, measuring oxidative stress in the hydrophilic and hydrophobic phases, showed similar results, making technical errors unlikely. Finally, echocardiography could have misclassified patients, despite using standard criteria [50].

In conclusion, we did not find evidence of systemic RAS activation or oxidative stress in patients with early HFpEF. This finding is consistent with the lack of efficacy of RAS inhibitors in the treatment of HFpEF. The lack of RAS activation and systemic oxidation seems to differentiate HFrEF from HFpEF. This suggests different mechanisms in genesis or propagation of these two forms of HF, which would explain the difference in benefit with treatment modalities.

## Figures and Tables

**Table 1:** Clinical characteristics of the study population.

	Cases (*N* = 25)	Control (*N* = 25)	*P* value
Demographic variables			
Age	64.8 ± 10.8	65.0 ± 11.3	1
Gender			**0.04**
Females	7 (28%)	14 (56%)	
Males	18 (72%)	11 (44%)	
Race^*∗*^			0.8
White	13 (54%)	12 (48%)	
Black	11 (46%)	12 (48%)	
Clinical variables			
Smoking	10 (40%)	10 (40%)	1
Diabetes	8 (32%)	9 (36%)	1
BMI	29.6 ± 4.8	25.3 ± 4.7	**0.00**
Hypertension	16 (64%)	14 (56%)	0.6
Mean SBP (mm Hg)	135.4 ± 19.2	127.4 ± 16.7	0.1
Mean DBP (mm Hg)	75.8 ± 13.0	74.0 ± 10.3	0.6
Hypercholesteremia	14 (56%)	10 (40%)	0.4
Medications			
Betablocker	15 (60%)	10 (40%)	0.3
ACEI	12 (48%)	9 (36%)	0.6
ARB	4 (16%)	1 (4%)	0.4
Diuretic	4 (16%)	8 (32%)	0.3
Statin	14 (56%)	16 (64%)	0.8

^*∗*^1 (4%) Asian in each group; HFpEF: heart failure with preserved ejection fraction; DD: diastolic dysfunction; BMI: body mass index; SBP: systolic blood pressure; DBP: diastolic blood pressure; ACEI: angiotensin converting enzyme inhibitor; ARB: angiotensin II receptor blocker.

**Table 2:** Oxidative stress markers and ACE activity in study population.

	Cases	Controls	*P* value
	Mean ± SD	Mean ± SD	
Oxidative stress measures			
(*E*_*h*_) CyS^*∗*^	70.1 ± 7.8	50.3 ± 11.6	**0.001**
(*E*_*h*_) GSH^*∗*^	118.8 ± 14.0	118.4 ± 16.5	0.9
DROMs^±^	375.2 ± 132.4	474.5 ± 167.5	**0.02**
IsoP^€^	15 × 10^2^ ± 6 × 10^2^	73 × 10^2^± 32 × 10^2^	**0.03**
ACE measures			
ACE-HHL^‡^	42.6 ± 9.6	36.8 ± 9	0.1
ACE-ZPHL^‡^	39.0 ± 8.7	36.2 ± 8.9	0.4
ACE PROT^§^	134.5 ± 38.2	101.9 ± 22.2	**0.03**

^**∗**^mV; ^±^Carr units; ^ €^pg/mL; ^‡^mU/mL; ^§^percentage (%); DD: diastolic dysfunction; *E*_*h*_ CyS: redox potential of reduced to oxidized cysteine (negative); *E*_*h*_ GSH: redox potential of reduced to oxidized glutathione (negative); DROMs: derivatives of reactive oxygen metabolites; IsoPs: isoprostanes; ACE-HHL: angiotensin converting enzyme activity measured using Hip-His-Leu (HHL) substrate; ACE-ZPHL: angiotensin converting enzyme activity measured using Z-Phe-His-Leu (ZPHL) substrate; ACE PROT: angiotensin converting enzyme protein levels.
